# Antimicrobial and cytotoxic activity of electrosprayed chitosan nanoparticles against endodontic pathogens and Balb/c 3T3 fibroblast cells

**DOI:** 10.1038/s41598-021-04322-4

**Published:** 2021-12-29

**Authors:** Amir Ibrahim, Desigar Moodley, Cosmas Uche, Ernest Maboza, Annette Olivier, Leslie Petrik

**Affiliations:** 1 Department of Restorative Dental Sciences, Faculty of Dentistry, Nile University, Khartoum North, Khartoum Sudan; 2 Department of Restorative Dentistry, Faculty of Dentistry, University of the Western Cape, Cape Town, South Africa; 3 Department of Chemistry, Faculty of Natural Sciences, University of the Western Cape, Cape Town, South Africa; 4 Department of Environmental Management, Federal University of Technology, PMB 1526, Owerri, Nigeria; 5 Oral and Dental Research Laboratory, Faculty of Dentistry, University of the Western Cape, Cape Town, South Africa

**Keywords:** Pulpitis, Antimicrobials, Applied microbiology, Biofilms, Nanoscale materials

## Abstract

The aims of this study were to synthesize highly positively charged chitosan nanoparticles (Ch-Np) using the electrospraying technique, and to test their antimicrobial activity against endodontic pathogens, and cytotoxicity against fibroblast cells. Ch-Np were synthesized from low molecular weight chitosan (LMW-Ch) using the electrospraying technique, and characterized. The antimicrobial activity was evaluated against *Streptococcus mutans*, *Enterococcus faecalis*, and *Candida albicans* in their planktonic state using a Time-Kill Test performed by using broth micro-dilution technique, and against biofilm biomass using a microtiter plate biofilm assay. The cytotoxicity was evaluated using Balb/c 3T3 fibroblast cells with the standard MTT assay. Electrospraying of LMW-Ch produced Ch-Np with an average size of 200 nm, and a surface charge of 51.7 mV. Ch-Np completely eradicated *S. mutans* and *E. faecalis* in the planktonic state and showed fungistatic activity against *C. albicans*. Furthermore, it significantly reduced the biofilm biomass for all the tested microbial species [*S. mutans* (*p* = 0.006), *E. faecalis* (*p* < 0.0001), and *C. albicans* (*p* = 0.004)]. When tested for cytotoxicity using 3T3 cells, Ch-Np showed no cytotoxicity. In conclusion, the highly positively charged, colloidal dispersion of Ch-Np are effective as a biocompatible endodontic antimicrobial agent.

The main cause of endodontic and periapical diseases is microbial contamination of the root canal system. This microbial contamination may range from bacterial, fungal to viral^1,2^. Thus, the aim of treating root canal infection is to eliminate microbial contamination from the entire root canal system^3^. Different treatment modalities are used to disinfect the root canal system, such as mechanical removal of the infected dentine^4^ in combination with antimicrobial agents in the form of root canal irrigants and intra-canal medicaments^5^. Other treatment modalities used to disinfect the root canal system include using laser therapy^6^, photodynamic therapy^7^, and ozone^8^.

However, one of the main shortcomings of the current root canal treatment modalities is the inability to completely eradicate persistent pathogens. For instance, nickel titanium rotary instruments were found to result in variable unprepared surfaces and thus incomplete removal of all pathogens from the root canal system^9–11^. Furthermore, some microbial species such as *Enterococcus faecalis* were resistant to commonly used antimicrobial root canal irrigants and medicaments^12^. Similarly, photodynamic therapy resulted in only 80% reduction of resistant endodontic pathogens such as *Actinomyces israelii, Fusobacterium nucleatum, Porphyromonas gingivalis*, and *Prevotella intermedia*^13^. The use of different wavelengths in laser therapy to disinfect the root canal system also showed ineffective results^14^.

Antimicrobial nanoparticles have shown a promising effect against resistant pathogens due to their unique physio-chemical properties^15^. The antimicrobial activity of nanoparticles against different microorganisms was found to be different from its original bulk state^16^. Polymeric nanoparticles have gained significant interest as new antimicrobial agents due to their biocompatibility and ability to eradicate microbial species by multiple mechanisms^17^. Chitosan is a natural polysaccharide that showed some antimicrobial activity at the macroscale level^18^. Furthermore, the presence of chitosan in a nano-scale form enhances its antimicrobial properties^19^. Chitosan nanoparticles (Ch-Np) can be synthesized using either cross-linking (chemical or physical) or drying techniques^20^. The drawback of using chemical cross-linking methods is the risk of synthesizing toxic and chemically less stable nanoparticles due to the effect of different types of solvents or cross-linkers used^21^. The limitation of using the physical cross-linking method to produce Ch-Np is the tendency of the Ch-Np to aggregate upon the addition of another polymer to produce the nanoparticles^22^.

Electrospraying is an example of a drying technique that is used to synthesize various nanoparticles. It is a simple method based on the synthesis of solid nanoparticles from a liquid by an electrical force and hence the name electrospraying^23^. The mechanism of nanoparticle formation through electrospraying depends on the force that acts on the bulk liquid while passing through a needle at a constant rate under the influence of high electrical potential. The passage of the electrical current through a liquid droplet can create an electrical or “Coulomb” force inside the droplet^24^. This electrical force will act against the cohesive force of the droplet caused by the surface tension of the dissolving liquid. Nanoparticles are formed when the Coulomb force exceeds the cohesive force, resulting in the droplets breaking into small particles on a nano-scale level, while the solvent evaporates from the droplet^25^.

Thus, the aims of this study were firstly to synthesize highly positively charged Ch-Np using the electrospraying technique and determine their characteristics. The second aim was to test the antimicrobial activity of the synthesized Ch-Np against resistant endodontic pathogens in their planktonic state and against their biofilm biomass. The third aim was to evaluate the biocompatibility of the synthesized Ch-Np toward fibroblast cells.

The null hypothesis of this study was that, electrospraying of LMW-Ch would not produce highly positively charged Ch-Np, that would not possess antimicrobial activity against endodontic pathogens in the planktonic state, nor against their biofilm biomass. Furthermore, these particles would possess a cytotoxic effect when tested using fibroblast cells.

## Methods

### Preparation of chitosan solution

Low molecular weight chitosan (LMW-Ch) (Merck, Sigma Aldrich, Saint Louis, MO, USA) with 50–190 KDa and a 75–85% degree of deacetylation was used at a concentration of 6% by mass by dissolving it in 90% (v/v) Trifluoroacetic acid (TFA) ReagentPlus 99% (Merck, Sigma Aldrich, Saint Louis, MO, USA). The chitosan was allowed to disperse completely in TFA using a magnetic stirrer (Bibby Heated Magnetic Stirrer HB502, Sterilin, England) at 1000 rpm for 4 h at 50 °C.

### Synthesis of Ch-Np (electrospraying)

The commercial LMW-Ch dispersion was dispensed in a syringe filled with a 19 mm gauge needle. The syringe was fixed in a 33 DDS dual drive independent channel syringe pump (Harvard apparatus, Massachusetts, MA, USA) for electrospraying. The chitosan colloidal suspension was electrosprayed in droplets at room temperature and a flow rate of 0.4 mL/h. A 25 kV potential was applied between the needle and the collector (aluminum foil) using a high voltage generator. TFA was evaporated from droplets during the time of flight and the dry chitosan nanoparticles were collected upon an aluminum foil collector that was placed 12 cm away from the tip of the needle.

### Characterization of Ch-Np

#### Hydrodynamic particle size and surface charge

The hydrodynamic size and surface charge of LMW-Ch and the electrosprayed Ch-Np were evaluated using the dynamic light scattering (DLS) method using Zetasizer Nano instrument, Nano S90 (Malvern Instruments, England).

The samples were prepared by suspending 0.005 g of LMW-Ch or electrosprayed Ch-Np in 1 mL of deionized water. The samples were vortexed for 2 min to assure complete dispersion and distribution of the materials before testing. For the analysis, 1 mL of each sample was placed in a UV-transparent disposable low volume cuvette. The scattered light was collected at an angle of 90° at 25 °C. The measurements were run in triplicate.

#### Scanning electron microscopy (SEM)

A small amount of the synthesized Ch-Np powder (experimental group) and LMW-Ch (control group) was placed on a stub coated with carbon, and the samples were then coated with a thin gold film. SEM images were taken at different magnifications and at various points of the samples using a Zeiss Gemini Auriga Scanning Electron Micro analyser, equipped with a CDU-led detector at 3.00 kV with a tungsten filament to determine the morphology and particle size of the electrosprayed Ch-Np.

#### Fourier-transform infrared spectroscopy

The structural configuration of the electrosprayed chitosan was evaluated by Fourier-transform infra-red (FTIR) spectroscopy using a Spectrum 400 FTIR/FT-NIR Spectrophotometer equipped with a universal ATR sampling accessory by PerkinElmer (PerkinElmer, Lantrisant, United Kingdom) to confirm the presence of the main functional groups of chitosan.

A drop of TFA and 0.1 g of the electrosprayed chitosan, LMW-Ch were placed on the sample holder (crystal) of the PerkinElmer Spectrum 400 FTIR/FT-NIR Spectrophotometer. Each of the samples was evaluated separately by collecting the signals in 32 scans of the infrared spectra within a range of 4000–650 cm^-1^ in transmittance (%) at room temperature.

### Antimicrobial assays

The antimicrobial activity was evaluated against planktonic cells of microorganisms related to root canal infection, namely *Streptococcus mutans* (ATCC 25175) (American Type Culture Collection, Manassas, VA, USA), *Enterococcus faecalis* (ATCC 29212), and *Candida albicans* (ATCC 90028). All microbial species were incubated in brain heart infusion broth (BHI) (Merck, Sigma Aldrich, Saint Louis, MO, USA) at 37 °C for 24 h. Cells from each microbial species were then suspended in phosphate buffer saline solution (PBS) (Merck, Sigma Aldrich, Saint Louis, MO, USA) and their concentrations were adjusted to 0.5 McFarland standard (Mcf) using DensiCHEK Plus (BioMérieux, Durham, NC, USA).

### Assessment of antimicrobial activity of LMW-Ch

The first step was to determine the antimicrobial activity of LMW-Ch against planktonic cells of *S. mutans*, *E. faecalis*, and *C. albicans*. The commercially supplied LMW-Ch was prepared into two concentrations, namely 1% and 3%, by dispersing 10 mg and 30 mg of chitosan, respectively, in 1 mL of 3% (v/v) acetic acid^26^. The selection of these two concentrations was chosen as the concentration of the chitosan increased the viscosity of the hydrogel beyond 3%^27^, which rendered it difficult to utilize.

The antimicrobial activity was evaluated using a Time-kill Test performed using the broth microdilution technique. A volume of 100 µL of each chitosan solution (1% or 3%) was dispensed in a sterile well of a 12-well cell culture plate, to which 200 µL of 0.5 (Mcf) from each tested microbial species suspension and 1700 µL of brain heart infusion broth (BHI) as a growth medium was added. The effect of acetic acid was evaluated as control by dispensing 100 µL of the acetic acid instead of the chitosan suspension. This was labelled as a negative control, while each microorganism’s normal growth rate was considered a positive control group. Each group was incubated at 37 °C for 24 h. At zero minutes, 30 min, 1, 2, 4, 6, 8, and 24 h, 100 µL was removed from each group, serially diluted and plated onto freshly poured brain heart infusion agar plates. After a 24 h incubation period, the number of colony forming units (CFU) in each plate were counted using an automated colony counter (Gerber, Lyss, Switzerland). The number of colony forming units that exceeded 300 were considered as too numerous to count (TNTC) and recorded as 300 (CFU), while those < 30 were considered as too low to count (TLTC) and recorded as zero and considered as insignificant to produce illness^28^. The test was repeated in triplicate (3 independent experiments on separate days, each with three repeats for each organism) following the clinical and laboratory standard institute standards for dilution antimicrobial susceptibility testing^29^.

### Assessment of antibacterial activity of Ch-Np

After establishing a baseline for the antimicrobial activity of LMW-Ch against endodontic pathogens, the second step was to evaluate and compare the antimicrobial activity of Ch-Np against planktonic cells. Furthermore, the effect of Ch-NP was evaluated against the biofilm biomass of the microbial species.

The synthesized Ch-Np were allowed to completely disperse as colloids in distilled water to form a final concentration of 3% (w/v). The concentration was selected to be comparable to the maximum concentration used in LMW-Ch. The mixture was placed in a 3 mL microtube and mixed using an Eppendorf Thermomixer 5350 Mixer (Marshall Scientific, Hamburg, Germany) for 10 min at 700 rpm to allow complete dispersion.

#### Antimicrobial activity of Ch-Np on planktonic microbial cells

The antimicrobial activity of Ch-Np was evaluated against planktonic cells of *S. mutans*, *E. faecalis*, and *C. albicans* using the same method as was used to evaluate the antimicrobial activity of LMW-Ch (Time-Kill test performed by the broth micro dilution technique). The results were compared to the antimicrobial activity of LMW-Ch.

#### Antimicrobial activity of Ch-Np on biofilm biomass

The effect of the Ch-Np against the biofilm biomass of the microbial species was evaluated using a microtiter plate biofilm assay. Biofilms of *S. mutans*, *E. faecalis*, and *C. albicans* were allowed to grow in sterile 96 well microtiter plates by plating 50 µL of 0.5 Mcf standard of each microbial species with 150 µL of BHI for 72 h at 37 °C. The BHI was discarded, and the biofilm was washed five times with sterile phosphate buffer saline (PBS) to remove any planktonic microbial cells and unattached microorganisms. In each of the 12 wells (n = 12), 50 µL of the prepared Ch-Np solution was added, along with 150 µL BHI, and incubated at 37 °C for 24 h. In the control group, Ch-Np was replaced with PBS. After 24 h, the BHI was removed, and each well was filled with 0.1% crystal violet to allow staining of the remaining microbial species’ biofilms for 10 min. The crystal violet was removed from the biofilm by adding 30% (v/v) acetic acid in each well to solubilize the crystal violet before measuring the optical density of each sample at a wave length of 540 nm using a microplate reader (Rayto, Germany).

### Cytotoxicity assay

The Balb/c 3T3 mouse fibroblast cells line (The National Repository for Biological Materials, Sandringham, Gauteng, South Africa) was used for the cytotoxicity assay. The cells were grown as described by Grobler et al*.*^30^. The cells were incubated under standard conditions at 37 °C, in 5% carbon dioxide and at 95% humidity in Dulbecco’s modified Eagles medium (DMEM). The medium was mixed with 1% penicillin/streptomycin mix (Cambrex Bio Science, Baltimore, MD, USA) and 10% fetal bovine serum as a supplement. The cells were sub-cultured every 48 h using trypsin 0.25% solution (HyClone, GE Health care, Life Science, South Logan, UT, USA).

The cells were grown to near confluency, and trypsinized, and subsequently diluted to a final suspension containing approximately 3 × 10^5^ cells/mL. A volume of 100 µL of suspension was plated in 96-well plates and allowed to attach to the well surface for 24 h and reach a strong growth phase.

The DMEM medium was replaced by 100 µL of LMW-Ch and Ch-Np and then incubated for 24 h. The control group contained Balb/c 3T3 cells and DMEM medium only. The survival rate of Balb/c 3T3 mouse fibroblast cells was evaluated using the MTT colorimetric assay as described by Mosmann^31^. This widely used assay is a sensitive, qualitative and most reliable colorimetric test that measures cells viability, proliferation and activation. Thereafter, 5 mg of MTT (3-(4,5-dimethylethylthiazol-2-yl)-2,5-diphenyl tetrazolium bromide (Merck, Sigma Aldrich, Saint Louis, MO, USA) was dissolved in 1 mL of PBS and sterilized. In each well of each group, 10 µL of the MTT was added and incubated at 37 °C. After 3 h, the medium that contained the MTT was discarded in all groups. To solubilize the Formazan crystals, 100 µL of di-methylsulfoxide (DMSO) was added to each well. The colour change was then measured as represented by the optical density of the living cells when absorbed at a wavelength of 540 nm using a microplate reader (Rayto Rt-2100C, Shenzhen, China). The assay was repeated four times (4 independent experiments on separate days).

### Data analysis

To evaluate the antimicrobial activity of LMW-Ch and Ch-Np against planktonic microbial cells, all results for each group were transferred to an Excel spreadsheet (Microsoft Corporation 2016, USA). The data was expressed as a mean of Log CFU/mL and then analyzed for the change in their mean Log CFU/mL numbers over time. Comparison between the effect of 1% and 3% LMW-Ch and between the effect of 3% Ch-Np and 3% LMW-Ch among each microbial species was tested using the Breslow (Generalized Wilcoxon) test.

To evaluate the effect of Ch-Np on biofilm biomass of *S. mutans*, *E. faecalis*, and *C. albicans* the data was expressed in optical density values and then analyzed using IBM SPSS statistical software (version 25; IBM, Armonk, NY, USA). The effect was evaluated by comparing the optical density of each microbial species biofilm before and after their exposure to Ch-Np. A Mann–Whitney *U* test was used to determine a statistically significant difference, if any, between the groups. A *p*-value of 0.05 was considered as a significant statistical difference.

Regarding the cytotoxicity assay, the results for each group were transferred to an Excel spreadsheet. The data were expressed as a mean of optical density values and then analyzed using IBM SPSS statistics software. The mean of each group was compared to the control group and expressed as a percentage of the control, which represents 100%. A *t* test was used to evaluate the statistical difference between each group and the control group. Pairwise comparison between each experimental group to their control was also analyzed using the Tukey pairwise post-hoc test to determine whether there was a statistical difference between the mean of all possible pairs using a studentized range distribution.

## Results

Electrospraying of LMW-Ch dispersed in TFA at room temperature with a flow rate of 0.4 mL/h and 25 kV potential resulted in the deposition of a layer of nanoparticles upon the collectors that was placed 12 cm away from the tip of the needle and perpendicular to it, which were collected and further characterized using the following characterization methods.

### Characterization of the synthesized Ch-Np

#### Hydrodynamic particle size and surface charge

**Figure 1:**
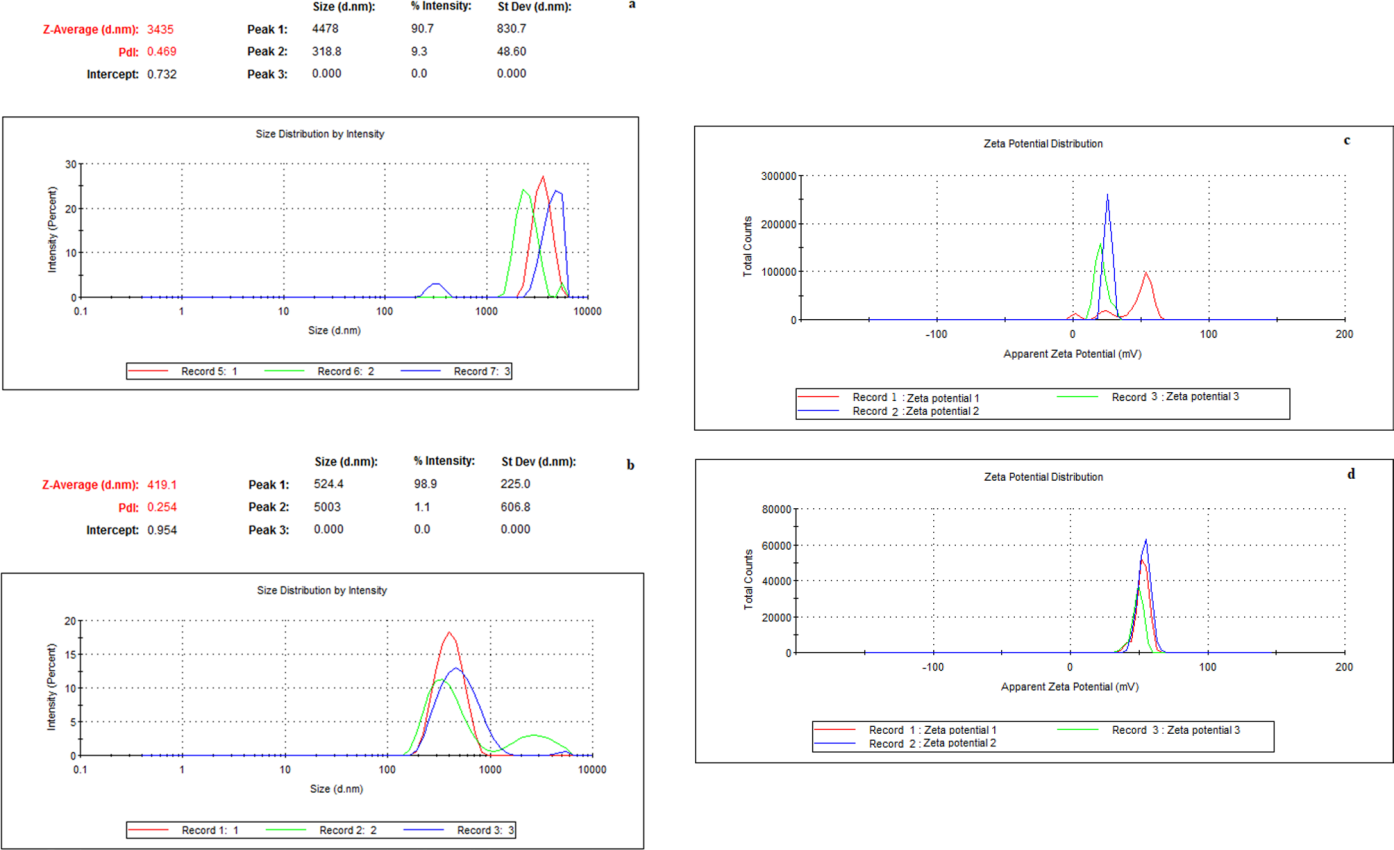
The average distribution of (**a**) hydrodynamic particle size and polydispersity index value of LMW-Ch (**b**) hydrodynamic particle size and polydispersity index value of Ch-Np, (**c**) zeta potential of LMW-Ch (**d**) zeta potential of Ch-NP as measured by Zetasizer Nano instrument.

The average hydrodynamic particle size distribution (Zetasizer) of the commercially supplied LMW-Ch was 3435 nm. Following dispersion of LMW-Ch in TFA and electrospraying, thus forming Ch-Np, the particle size was decreased by an order of magnitude to an average size of 419 nm when the dried particles were suspended in water for the particle size measurement (Fig. 1a, b). The measured surface charge (zeta potential) of suspended LMW-Ch particles was 25.6 mV. Following electrospraying and formation of Ch-Np, this value increased to 53.7 mV, indicating that highly positively surface charged nanoparticles had formed (Fig. 1c, d). The average polydispersity index value of LMW-Ch was 0.469, while that of the synthesized Ch-Np was 0.254 (Fig. 1a, b), showing their greater uniformity.

#### Scanning electron analysis

**Figure 2:**
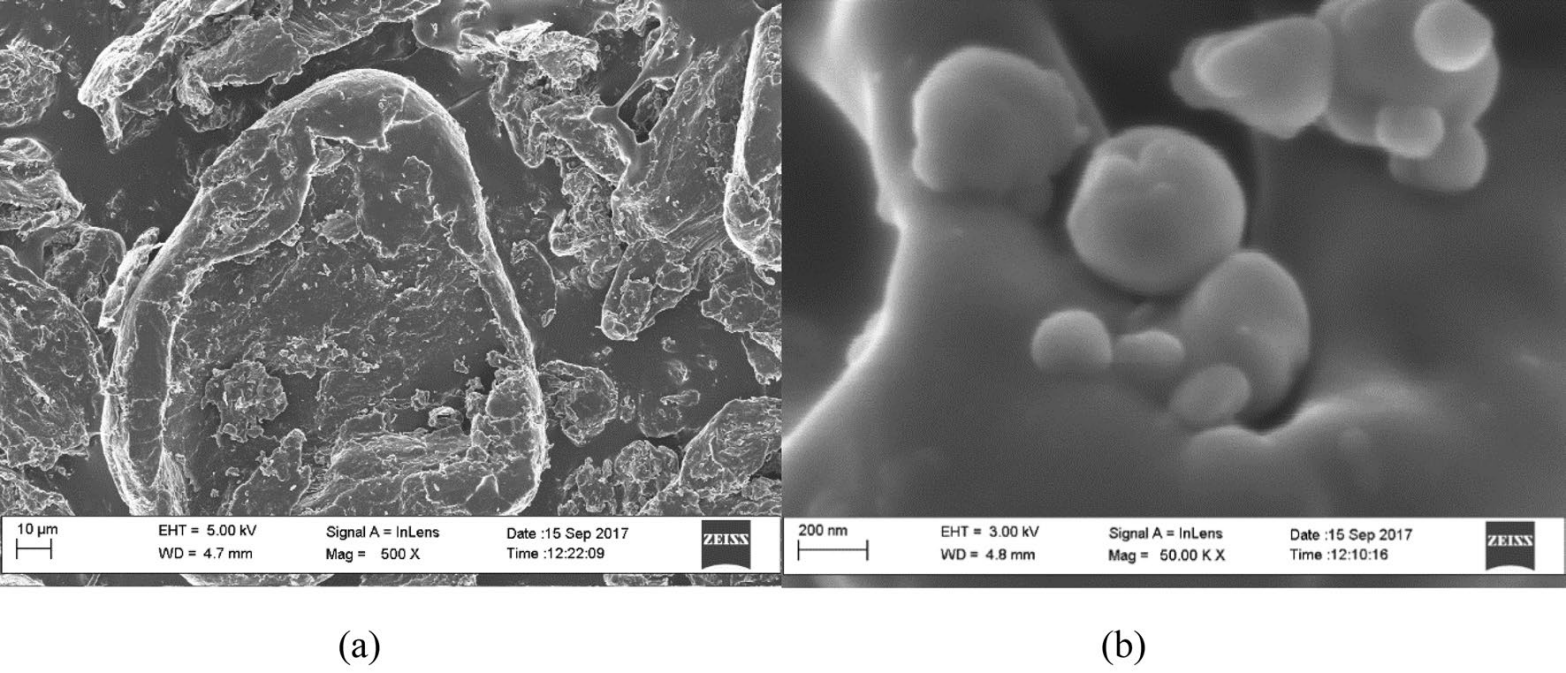
HR-SEM of (**a**) LMW-Ch and (**b**) Ch-Np synthesized using electrospraying.

The HR-SEM of the LMW-Ch and the electrosprayed Ch-Np at different magnifications showed a reduction in the particle size of LMW-Ch from an average greater than 10 µm to a nano-scale level for Ch-Np with an average size of 200 nm and irregular shape (Fig. 2), which was smaller than the hydrodynamic particle size. Furthermore, the SEM showed the non-homogenous shape of Ch-Np with a higher tendency to form small agglomerates upon drying.

#### Fourier-transform infrared spectroscopy

**Figure 3:**
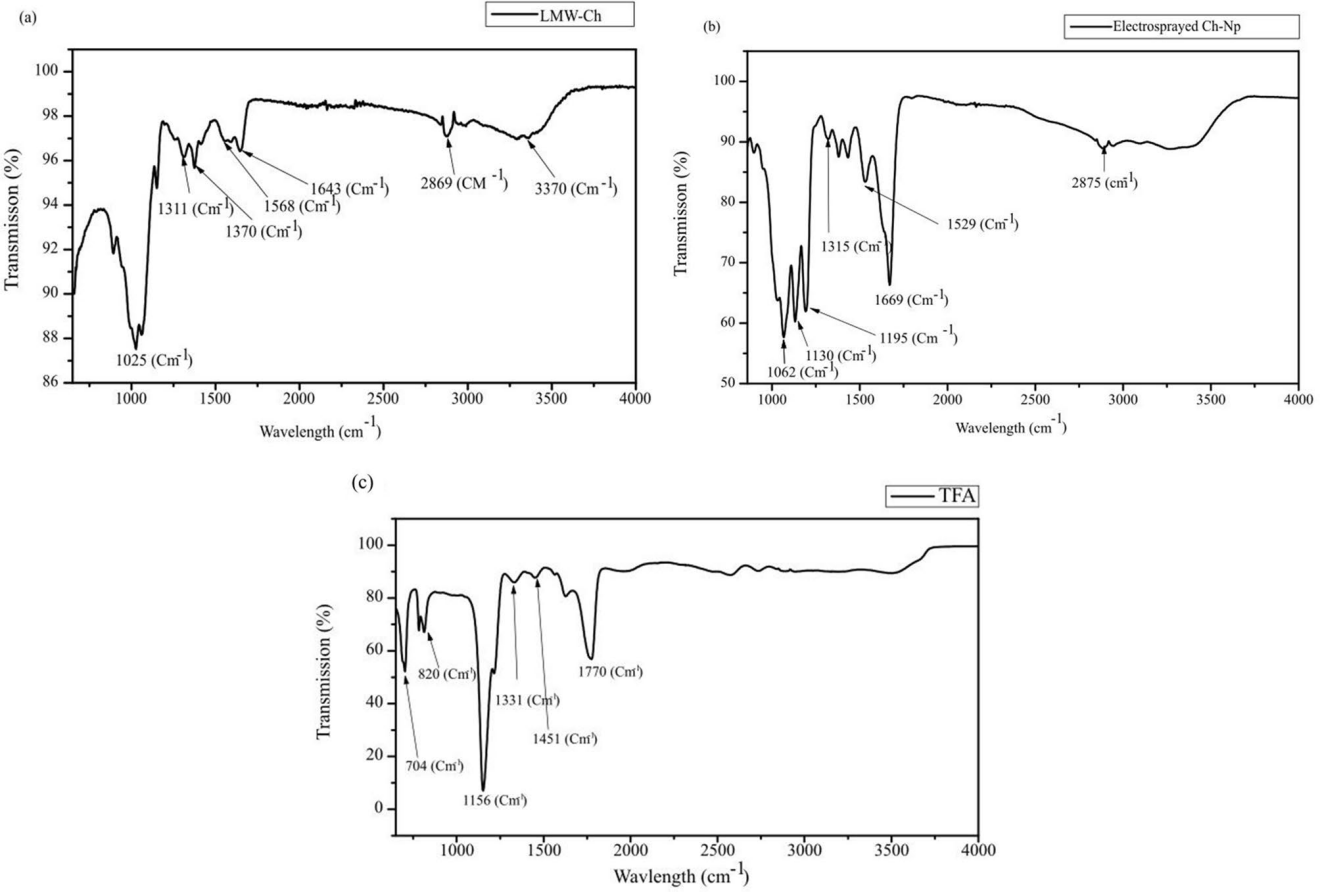
FTIR spectrum of (**a**) the LMW-Ch, (**b**) electrosprayed Ch-Np and **(**c) TFA showing the transmission peak of different functional groups.

The FTIR analysis of LMW-Ch was conducted to identify the functional groups present on the surface of the LMW-Ch. The FTIR spectrum of LMW-Ch (Fig. 3a) showed a vibration with a peak at 1643 cm^−1^ which represents the carbonyl group (C=O) and stretching of the secondary amide (amide I), while the peak at 1568 cm^−1^ was assigned to the N–H bend and CH_3_ stretch (amide II). The CH_2_ wagging (C–N vibration from amide) amide III functional group was identified at 1311 cm^−1^. Similar peaks positions for chitosan were reported^32^.

Similar peaks were observed on the FTIR of the electrosprayed chitosan (Ch-Np) that showed an absorption peak at 1669 cm^−1^ (Fig. 3b), which was assigned to the carbonyl group (C=O) vibration and stretching of the secondary amide (amide I). The peak at 1529 cm^−1^ was assigned to N–H bend, C–N stretch (amide II), while the peak at 1315 cm^−1^ was assigned to CH_2_ bend, CH_3_ symmetric distortion (amide III). The peak at 1130 cm^−1^ was assigned to asymmetries in the phase ring stretch mode, while the peak at 1062 cm^−1^ was assigned to C–O–C symmetric stretch in phase (glucosamine) ring. Those peaks were in the same range of amide I, amide II, and amide III, respectively, confirming that the newly formed particles (Ch-Np) were similar to chitosan. Furthermore, the intense sharp peak at 1669 cm^−1^ which was assigned to the trifluoroacetyl ester group, indicated the ability of TFA to form an amine salt (trifluoroacetyl ester) with the amine group of the chitosan. The formation of the trifluoroacetyl ester group was also reported in the literature when chitosan was dispersed in TFA^33^.

The obtained FTIR spectrum of the TFA itself (Fig. 3C) showed a sharp peak at 1770 cm^−1^ which was assigned to C=O stretch, while the peak at 1451 cm^−1^ was assigned to C–O stretch. The peak at 1331 cm^−1^ was assigned to the HOC group. The high sharp peak at 1156 cm^−1^ was assigned to the CF_3_ group. The peaks at 820 cm^−1^ and 704 cm^−1^ were assigned to O–C=O stretching. Those peaks were not observed on the FTIR of the electrosprayed Ch-Np, and thus confirm that TFA was completely evaporated during the electrospraying process.

### Assessment of antibacterial activity of Ch-Np

Since the formation of the nano-scale size and highly positively charged nature of Ch-Np was confirmed by the Zetasizer, the second step was to evaluate the antimicrobial activity of Ch-Np against some of the resistant endodontic microbial species. The first step was to establish a baseline antimicrobial activity of the LMW-Ch, which was then compared to the antimicrobial activity of the synthesized Ch-Np.

### Antimicrobial activity of LMW-Ch and Ch-Np against planktonic cells

**Figure 4:**
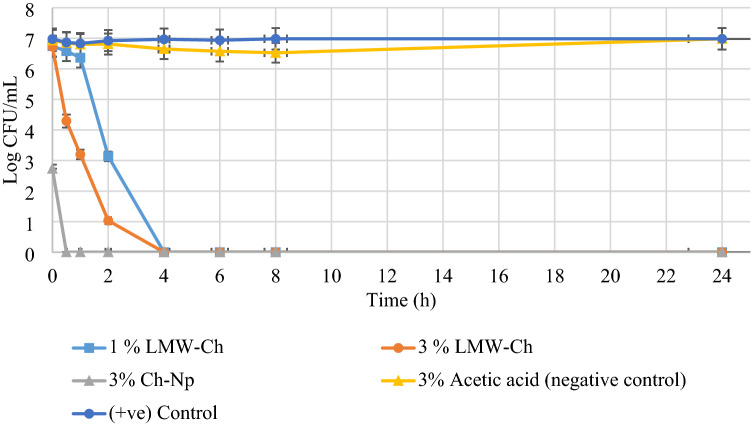
The mean Log CFU/mL of *S. mutans* following its exposure to 1%, 3% LMW-Ch and 3% Ch-Np at different time intervals.

Using 3% acetic acid (negative control group) as a solvent of LMW-Ch did not alter the growth of *S. mutans*, *E. faecalis*, and *C. albicans*, as the mean number of the Log CFU/mL was not reduced to zero during all the times tested (Figs. 4, 6, 7), thus excluding any antimicrobial effect of using 3% acetic acid as a solvent for LMW-Ch.

Both concentrations of LMW-Ch (1% and 3%) showed bactericidal action against *S. mutans* and *E. faecalis*. However, 3% LMW-Ch showed more potent antimicrobial activity against both microbial species than the 1% concentration. For instance, *S. mutans* was completely eradicated at 4 h contact time when either of the concentrations of LMW-Ch was used, although 3% LMW-Ch showed greater reduction in the mean Log CFU/mL over time compared to 1% LMW-Ch. However, using 3% Ch-Np showed complete eradication of *S. mutans* at 30 min contact time (Fig. 4).

**Figure 5:**
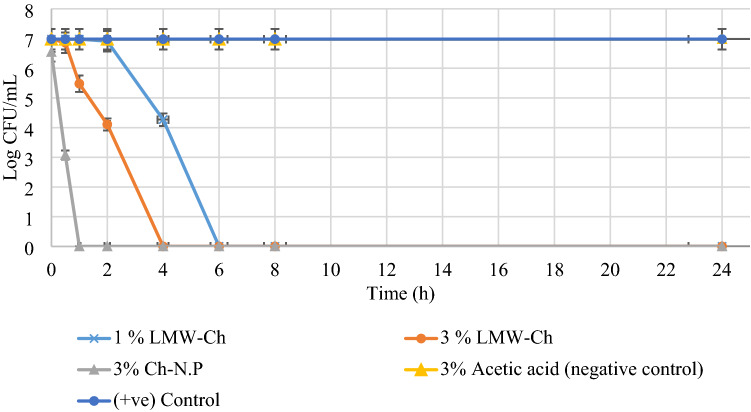
The mean Log CFU/mL of *E. faecalis* following its exposure to 1%, 3% LMW-Ch and 3% Ch-Np. The number of the Log CFU/mL of the two control groups grow at the same rate hence the two lines in the graph were superimposed.

*E. faecalis* was completely eradicated after 6 h of contact time following exposure to 1% LMW-Ch (Fig. 5). This time was reduced to 4 h contact time when the concentration of LMW-Ch was increased to 3%. However, *E. faecalis* was completely eradicated at 1 h contact time following exposure to 3% Ch-Np.

**Figure 6:**
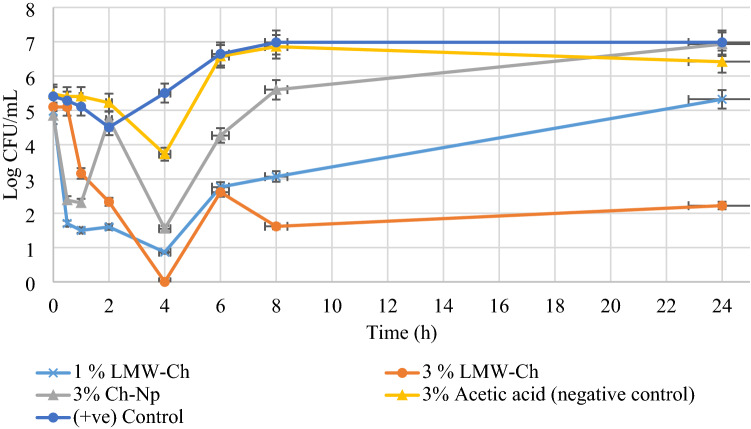
The mean Log CFU/mL of *C. albicans* following its exposure to 1%, 3% LMW-Ch and 3% Ch-Np.

*C. albicans* showed erratic behavior following exposure to LMW-Ch (Fig. 6). Using 1% LMW-Ch could not eradicate *C. albicans* after 24 h contact time. However, at 3% LMW-Ch *C. albicans* was not observed at 4 h contact time but re-emerged again at 6 h and continued to be observed up to 24 h. Furthermore, using 3% Ch-Np could not eradicate *C. albicans* either, although there was a decline from 1 to 4 h and re-emergence in the mean Log CFU/mL thereafter at different time intervals, showing the ability of planktonic cells of *C. albicans* to resist the antimicrobial properties of Ch-Np after the initial decline. This would show that there is a window period during which *C. albicans* is weakened and unable to propagate for about 4 h when exposed to 3% Ch-Np and LMW-Ch, which was further verified by a statistical approach.

Comparison between the effect of 1% and 3% LMW-Ch among each microbial species was tested using the Breslow (Generalized Wilcoxon) test. The results of the three tests showed no statistical difference between using 1% and 3% LMW-Ch as an antimicrobial agent against *S. mutans*, *E. faecalis*, and *C. albicans* (*p* > 0.05) (Supplementary Table S1).

Furthermore, a comparison between the effect of 3% Ch-Np and 3% LMW-Ch among each microbial species was tested using the Breslow (generalized Wilcoxon) test. The Breslow test showed a statistical difference between the effect of 3% Ch-Np and 3% LMW-Ch against *E. faecalis* (*p* < 0.05). There was no statistical difference between the effect of 3% Ch Np and 3% LMW-Ch against *S. mutans*, and *C. albicans* (*p* < 0.02) (Supplementary Table S2).

### Antimicrobial activity of Ch-Np on biofilm biomass

**Figure 7:**
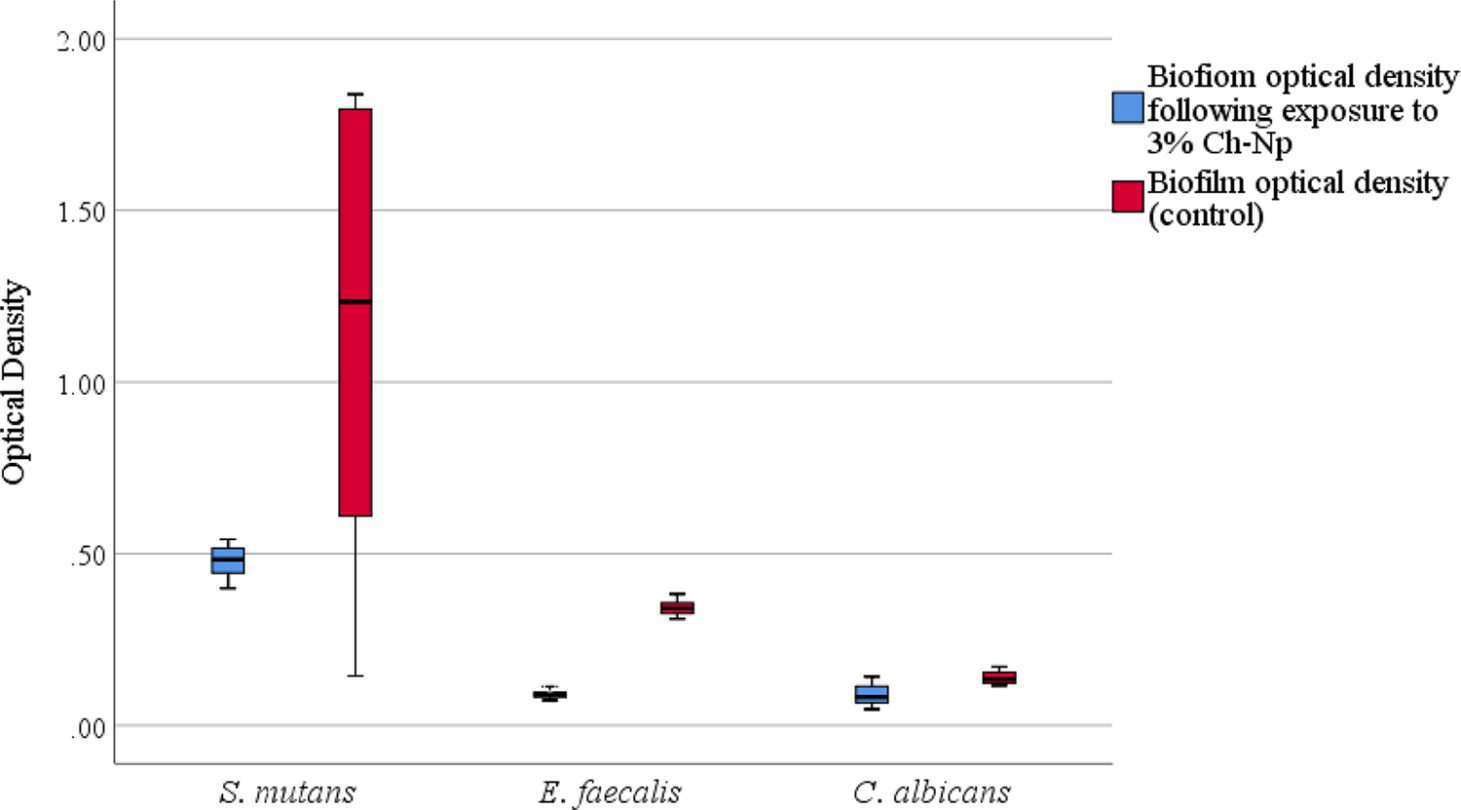
Box and Whisker plots demonstrating the median, distribution, maximum and minimum values of the biofilm optical density of *S. mutans*, *E. faecalis* and *C. albicans* in normal conditions (control) and following exposure to 3% Ch-Np. The vertical bars represent the lower and higher optical density values of the remaining microbial biofilm.

Using 3% Ch-Np as an antimicrobial agent showed greater antimicrobial activity against *S. mutans* and *E. faecalis* compared to 3% LMW-Ch and also an initial fungistatic effect against *C. albicans*. Thereafter, the antimicrobial activity of Ch-Np against biofilm biomass of the three microbial species was evaluated. The mean, median and standard error of the optical density of each microbial species’ biofilm in a control condition and following its exposure to 3% Ch-Np are shown in Supplementary Table S3. There was a difference in the distribution of the biofilm optical density of *S. mutans*, *E. faecalis*, and *C. albicans* before and after exposure to 3% Ch-Np (Fig. 7). The Y-axis in Fig. 7 represents the optical density values of each microbial biofilm. These values are not represented in percentages but are represented as a number that ranges from a minimum of zero to a maximum of 2.

3% Ch-Np could reduce the biofilm biomass of the three tested pathogens compared to their control, which was statistically significant (*S. mutans* (*p* = 0.006), *E. faecalis* (*p* = 0.0001), and *C. albicans* (*p* = 0.004)) when analyzed using the Mann Whitney U test (Supplementary Table S4).

### Cytotoxicity assay

**Figure 8:**
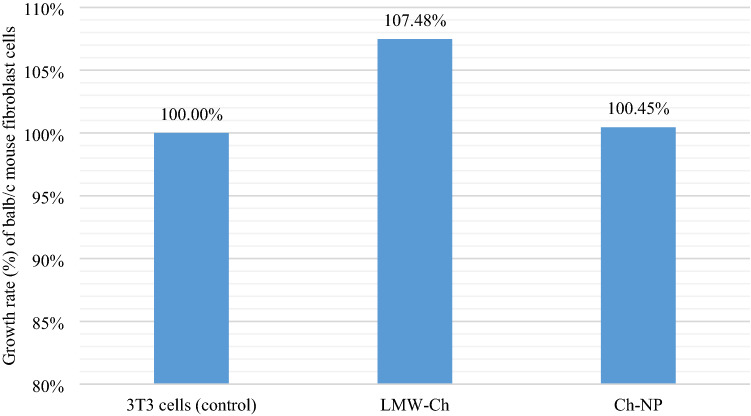
The growth rate of Balb/c 3T3 mouse fibroblast cells when exposed to LMW-Ch and Ch-Np compared to the control condition.

The Balb/c 3T3 fibroblast cells showed a mean optical density value of 0.69 and hence 100% growth rate in the control condition (Supplementary Table S5). This value increased to 0.74 following its exposure to LMW-Ch and hence increased the growth rate by 7.48% (Fig. 8), indicating no cytotoxicity but rather cell proliferation. Moreover, following exposure of Balb/c 3T3 mouse fibroblast cells to Ch-Np, the mean of the optical density values of the Balb/c 3T3 mouse fibroblast cells was similar to the control value, which was 0.67 with a slight increase in their growth rate from 100 to 100.45% (Fig. 8), indicating no cytotoxicity but rather biocompatibility.

A comparison was made between the mean growth rates of Balb/c 3T3 mouse fibroblast cells in each group using a *t* test to determine any statistically significant difference between the two groups. There was a statistical difference (*p* = 0.004) between the growth rate of 3T3 fibroblast cells in normal conditions and following their exposure to LMW-Ch. In comparison, Ch-Np showed no statistical difference (*p* = 0.89) between the growth rate of 3T3 fibroblast cells in normal conditions and following their exposure to Ch-Np (Supplementary Table S6).

For further refinement, Tukey pairwise post-hoc comparison was done to compare any statistical differences between the groups. Only two groups of LMW-Ch showed statistical differences from their control groups (Supplementary Table S7). However, using Ch-Np showed no statistical difference between the experimental groups and their control groups (Supplementary Table S8).

## Discussion

Using nanoparticles as an antimicrobial agent has increased recently in dental sciences due to their numerous advantages, such as their broad-spectrum antimicrobial activity^34^. In endodontics, the presence of some microbial species such as *E. faecalis*, *S. mutans*, and *C. albicans* is mainly associated with secondary root canal infection due to their ability to resist the commonly used endodontic antimicrobial agents^35^. In this study, the null hypothesis was rejected, because electrospraying resulted in highly positively charged Ch-Np that showed antimicrobial activity against endodontic pathogens in the planktonic state and against their biofilm biomass while maintaining their biocompatibility. The study showed that it was possible to prepare Ch-Np by electrospraying a commercially available LMW-Ch and applying the nanoparticles so formed, in an attempt to eradicate some endodontic microbial species.

LMW-Ch was used as a precursor to synthesize Ch-Np since the literature showed that the antimicrobial efficacy of chitosan was enhanced as the molecular weight decreased^36,37^. Highly positively charged Ch-Np were synthesized using the electrospraying technique. TFA as a solvent during electrospraying the chitosan was beneficial as it evaporated readily^33^, facilitating the rapid formation of dry Ch-Np and thus allowing deposition of dry nanoparticles at the collector. Furthermore, the electrospraying and evaporation process resulted in the deposition of irregularly shaped particles in the nano-scale range, as the Zeta size analysis showed. Similar particle shapes were obtained when different concentrations of acetic acid were used as a solvent for the preparation of Ch-Np using the electrospraying technique^38^. These results may reflect the reproducibility of similar-shaped Ch-Np when the electrospraying technique is used, despite the various solvents used.

The size of the supplied LMW-Ch was on average 3435 nm when suspended in water used for the Zetasizer measurement, which size was an order of magnitude greater than Ch-Np. The deposited Ch-Np particle size was 419 nm on average. Although the obtained size in this study was larger than that obtained by the ionic gelation technique^39^, it falls within the definition of polymeric nanoparticles (within the range of 10–1000 nm)^40^. Similar sizes were also obtained when an electrospraying technique was used with acetic acid as a solvent^41^. In this study, several factors may have contributed to the obtained particle size, such as the TFA solvent used, the needle diameter, the applied voltage, the distance between the needle tip and the collector, the flow rate, the concentration and thus the viscosity of the chitosan solution used.

The higher values of polydispersity of LMW-Ch (0.469) than that of Ch-Np (0.254) obtained in this study showed greater uniformity of Ch-Np. However, the fact was that both values were > 0.07 indicating the broad size distribution of both samples and thus the presence of agglomeration as observed by SEM analysis. The relation between the polydispersity index and the presence of agglomerated nanoparticles was described by the ISO 22412:2017.

The synthesized Ch-Np showed a higher zeta potential value (53.7 mV) compared to the LMW-Ch (25.6 mV). This increase in the surface charge value may be due to the ability of the Ch-Np to retain the electrical current that passes through the LMW-Ch droplet during electrospraying. The clinical importance of obtaining a higher zeta potential is that it will determine the affinity of the nanoparticles to the microbial cells, and it will influence its stability in a suspension^42^. Therefore, higher zeta potential can be expected to increase the antimicrobial properties of the synthesized Ch-Np, which may result in better antimicrobial activity than Ch-Np synthesized by other techniques.

The FTIR analysis of LMW-Ch was conducted to identify the functional groups present on the surface of the LMW-Ch. The main functional groups were amide I, amide II and amide III, which peaked at 1643 cm^−1^, 1568 cm^−1^ and 1311 cm^−1^, respectively (Fig. 3a). Similar peaks were also obtained when chitosan was produced by deacetylation of chitin extracted from silkworm chrysalides^32^. The FTIR of the electrosprayed Ch-Np showed that the three peaks, 1669 cm^−1^, 1529 cm^−1^ and 1315 cm^−1^ (Fig. 3b) were in the same range for amide I, amide II, and amide III respectively, confirming that the resulted nanoparticles formed in this study were Ch-Np. Furthermore, the intense sharp peak at 1669 cm^−1^ which was assigned to the trifluoroacetyl ester group, indicated the ability of TFA to form an amine salt (trifluoroacetyl ester) with the amine group of the chitosan. Furthermore, the presence of the trifluoroacetyl ester functional group in electrosprayed Ch-Np may facilitate its dispersion in water^33^. This is an important feature for the newly synthesized Ch-Np to be used as a root canal antimicrobial agent.

Since the formation of highly positively charged Ch-Np was confirmed, the second step was to evaluate its antimicrobial activity against some of the resistant endodontic microbial species. One of the critical issues regarding any root canal antimicrobial agent is the time required by those agents to eradicate the endodontic pathogens inside the root canal system (the contact time). In this study, the first step was to establish a baseline for the antimicrobial activity of the LMW-Ch in 1% and 3% concentrations. There was no statistical difference between using 1% LMW-Ch and 3% LMW-Ch regarding their antimicrobial efficacy. However, using 3% LMW-Ch showed better eradication time compared to using 1% LMW-Ch for the three tested pathogens. This is why 3% Ch-Np was used to compare its effect to the 3% LMW-Ch and evaluate the effect of size on its antimicrobial efficacy.

Using chitosan at a nano-scale level in this study showed enhanced antimicrobial activity against planktonic cells of *S. mutans* and *E. faecalis* compared to LMW-Ch regarding of the time required to eradicate those microbial species. This shows its potential to minimize the time required to eradicate these pathogens and thus reduce clinical chairside time. Although Ch-Np could be fungistatic, it did not eradicate *C. albicans*, which may be due to the rigidity of the fungal cell wall, which may prevent the penetration of Ch-Np into the fungal cell. Furthermore, the ability of planktonic cells of *C. albicans* to re-grow following their exposure to Ch-Np may be explained by the possibility of *C. albicans* to develop resistance to Ch-Np. Literature showed that *C. albicans* can adapt to environmental changes and produce genetically altered species for better adaptation to the new stressed environment^43^. This assumption is demonstrated in Fig. 6 as the mean number of Log CFU/mL that showed an initial reduction followed by an increase in the mean Log CFU/mL after 4 h. The fluctuation in the mean number of Log CFU/mL over time may indicate injury of some of the *C. albicans* cells, while other cells start to produce a new strain resistant to the effect of Ch-Np. Nevertheless, the fungistatic action of the Ch-Np would allow sufficient time for root canal irrigation work to be completed within 4 h in a relatively sterile environment.

The antimicrobial activity of the synthesized Ch-Np in this study, was comparable to the antimicrobial activity of Ch-Np synthesized using the ionic gelation method. However, in this study, *E. faecalis* was completely eradicated within 30 min’ contact time compared to 8 h contact time reported in prior studies^44,45^.

The presence of endodontic pathogens in a biofilm state enhances their pathogenicity and contributes to their resistance to commonly used endodontic antimicrobial agents such as sodium hypochlorite^46^. The synthesized Ch-Np resulted in a significant reduction in the biofilm biomass of the three tested pathogens, including *C. albicans*. This effect may be due to the ability of the nanoparticles to penetrate the biofilm’s extracellular polysaccharide matrix as a result of its small particle size and then to attach to the negatively charged microbial cell wall because of its highly positively charged surface. Similarly, other studies showed that the antimicrobial mechanism of nanoparticles against the integrity of microbial biofilm might be due to changes in the structural integrity and electrochemical interaction on the surface and in the enzymes of the microbial cells, following their penetration through the extracellular polymeric matrix^47,48^.

*E. faecalis* biofilm formation is enhanced by the presence of *S. mutans*^49^. As a result, the antimicrobial activity of Ch-Np shown in this study may further contribute to the elimination of *E. faecalis* through its antimicrobial effect against *S. mutans* and its ability to disrupt the biofilm biomass of the two pathogens. The significant reduction in the biofilm biomass formation of *C. albicans* following its exposure to Ch-Np may also enhance the antimicrobial effect of other endodontic antimicrobial agents that cannot penetrate the biofilm’s extracellular polysaccharide. The effect of Ch-Np to reduce the biofilm biomass was also observed when different techniques of nanoparticle synthesis were used^44,45^. In general, regarding the antimicrobial activity of the synthesized Ch-Np in this study, it showed a higher antimicrobial effect together with a shorter time required to eradicate planktonic microbial cells, which is one of the critical issues regarding any root canal antimicrobial agent.

The synthesized Ch-Np were not cytotoxic but did not increase the growth rate of the 3T3 mouse fibroblast cell line significantly, when compared to LMW-Ch and the control group, showing its biocompatibility when analyzed using a *t*-test. Similar effects were reported in this study when post-hoc analysis was used to analyze for any statistical difference between the experimental groups and their controls. However, when the post-hoc analysis compared the effect of LMW-Ch against 3T3 fibroblast cells to their controls, LMW-Ch showed no statistical difference in the two groups. The significant increase in the growth rate of 3T3 cells when exposed to LMW-Ch that was observed when the *t* test was applied may be due to the higher optical density values in the other two groups, which were statistically significant.

The negligible reduction in the growth rate of the 3T3 mouse fibroblast cells following their exposure to Ch-Np may be due to the reduction of the LMW-Ch molecular weight as a result of the change in its particle size, which in turn resulted in a reduction in its polydispersity index^45^. This result was advantageous compared to other results reported in the literature that showed the relatively more significant cytotoxic effect of typical root canal antimicrobial agents such as sodium hypochlorite and calcium hydroxide^50,51^.

One of the limitations of this study is that it did not evaluate the antimicrobial activity of the synthesized Ch-Np against mixed root canal pathogens in planktonic and biofilm state for an extended period of time to ensure the sustainability of the antimicrobial activity. Also, this study used a microtiter plate to evaluate the antimicrobial activity of Ch-Np. The use of a dentine model and other assessment methods to evaluate its antimicrobial activity, such as confocal laser microscopy, may provide additional information regarding the activity of Ch-Np.

## Conclusion

Highly positively charged chitosan nanoparticles can be synthesized using the electrospraying technique in the presence of trifluoroacetic acid as a solvent. Furthermore, the presence of the trifluoracetyl ester functional group in the electrosprayed Ch-Np may facilitate the solubility or high colloidal dispersion in water. The Ch-Np synthesized using this technique could eradicate some of the resistant endodontic pathogens in the planktonic state in a short period of time and reduce the biofilm biomass of these pathogens while maintaining its biocompatibility. Thus, Ch-Np synthesized with the electrospraying technique could be a potential endodontic antimicrobial agent that can be used in the form of a root canal irrigant or as an intra-canal medicament.

## Supplementary Information

Supplementary Tables.
