# Supplementary material for: Antimicrobial and cytotoxic activity of electrosprayed chitosan nanoparticles against endodontic pathogens and Balb/c 3T3 fibroblast cells
Source: Sci Rep. 2021 Dec 29;11:24487. doi: 10.1038/s41598-021-04322-4 (PMC8716534; doi:10.1038/s41598-021-04322-4)
Supplement: Supplementary file 1 — Supplementary Tables. [file 41598_2021_4322_MOESM1_ESM.docx]

**Title: Antimicrobial and cytotoxic activity of electrosprayed chitosan nanoparticles against endodontic pathogens and Balb/c 3T3 fibroblast cells**

Table S 1: Pairwise comparison between the antimicrobial effect of different LMW-Ch concentrations against *S. mutans*, *E. faecalis* and *C. albicans*.

|  | Microorganism | LMW-Ch concentration | 1% |  | 3% |  |
| --- | --- | --- | --- | --- | --- | --- |
|  |  |  | Chi-Square | Sig. | Chi-Square | Sig. |
| Breslow (Generalized Wilcoxon) | *S. mutans* | 1% |  |  | 2.83 | 0.09 |
|  |  | 3% | 2.83 | 0.09 |  |  |
|  | *E. faecalis* | 1% |  |  | 3.49 | 0.06 |
|  |  | 3% | 3.49 | 0.06 |  |  |
|  | *C. albicans* | 1% |  |  | 1.72 | 0.19 |
|  |  | 3% | 1.72 | 0.19 |  |  |

Table S 2: Pairwise comparison between the antimicrobial effect of 3% Ch-Np and 3% LMW-Ch against *S. mutans*, *E. faecalis* and *C. albicans*.

|  | Microorganism | Particle size | Ch-Np | | LMW-Ch | |
| --- | --- | --- | --- | --- | --- | --- |
|  |  |  | Chi-Square | Sig. | Chi-Square | Sig. |
| Breslow (Generalized Wilcoxon) | *S. mutans* | Ch-Np |  |  | 2.32 | 0.13 |
|  |  | LMW-Ch | 2.32 | 0.13 |  |  |
|  | *E. faecalis* | Ch-Np |  |  | 6.33 | 0.01 |
|  |  | LMW-Ch | 6.33 | 0.01 |  |  |
|  | *C. albicans* | Ch-Np |  |  | 0.35 | 0.56 |
|  |  | LMW-Ch | 0.35 | 0.56 |  |  |

Table S 3: The mean, median and standard error for the optical density of *S. mutans*, *E. faecalis* and *C. albicans* biofilm in normal conditions (control) and following exposure to 3% Ch-Np

|  | Mean | Median | Std. Error of Mean | Range | Skewness | Std. Error Skewness |
| --- | --- | --- | --- | --- | --- | --- |
| *S. mutans* | 0.48 | 0.48 | 0.01 | 0.14 | -0.28 | 0.64 |
| *S. mutans* (control) | 1.14 | 1.23 | 0.20 | 1.69 | -0.25 | 0.64 |
| *E. faecalis* | 0.09 | 0.09 | 0.00 | 0.04 | 0.69 | 0.66 |
| *E. faecalis* (control) | 0.34 | 0.34 | 0.01 | 0.07 | 0.27 | 0.64 |
| *C. albicans* | 0.09 | 0.08 | 0.01 | 0.10 | 0.72 | 0.66 |
| *C. albicans* (control) | 0.14 | 0.13 | 0.01 | 0.06 | 0.41 | 0.64 |

Table S 4: The effect of 3% Ch-Np on the biofilm biomass of *S. mutans*, *E. faecalis* and *C. albicans* (statistical analysis using the Mann-Whitney U test).

|  | *S. mutans* | *E. faecalis* | *C. albicans* |
| --- | --- | --- | --- |
| Z | -2.714 | -4.161 | -2.829 |
| Asymp. Sig. (2-tailed) | 0.007 | 0 | 0.005 |
| Exact Sig. [2*(1-tailed Sig.)] | .006b | .000b | .004b |

Table S 5: The mean, standard deviation and standard error of the mean of the optical density of Balb/c 3T3 mouse fibroblast cells in control condition and when exposed to LMW-Ch and Ch-Np.

|  |  | Mean | Std. Deviation | Std. Error Mean |
| --- | --- | --- | --- | --- |
| LMW-Ch | Experimental group | 0.74 | 0.10 | 0.01 |
|  | Control group | 0.69 | 0.10 | 0.01 |
| Ch-Np | Experimental group | 0.67 | 0.11 | 0.11 |
|  | Control group | 0.67 | 0.13 | 0.13 |

Table S 6: Comparison between the growth rate of the control of Balb/c 3T3 mouse fibroblast cells and LMW-Ch group using a t-test showing statistical difference between the two groups.

|  | Levene's Test for Equality of Variances | | t-test for Equality of Means | | | | | | |
| --- | --- | --- | --- | --- | --- | --- | --- | --- | --- |
|  | F | Sig. | t | degree of freedom | Sig. (2‑tailed) | Mean Difference | Std. Error Difference | 95% Confidence Interval of the Difference | |
|  |  |  |  |  |  |  |  | Lower | Upper |
| LMW-Ch | 0.58 | 0.45 | 2.95 | 126.00 | 0.00 | 0.05 | 0.02 | 0.02 | 0.09 |
| Ch-Np | 9.12 | 0.00 | 0.15 | 126.00 | 0.89 | 0.00 | 0.02 | -0.04 | 0.04 |

Table S 7: Tukey pairwise post-hoc comparison between the growth rate of the control of Balb/c 3T3 mouse fibroblast cells and LMW-Ch groups showing statistical difference between using two groups only.

|  |  |  | Mean Difference (I-J) | Std. Error | Sig. | 95% Confidence Interval | |
| --- | --- | --- | --- | --- | --- | --- | --- |
|  |  |  |  |  |  | Lower Bound | Upper Bound |
| Tukey HSD | Experimental group 1 | Control group 1 | .078813^*^ | .020670 | .005 | .01504 | .14258 |
|  | Experimental group 2 | Control group 2 | -.001375 | .020670 | 1.000 | -.06515 | .06240 |
|  | Experimental group 3 | Control group 3 | .029063 | .020670 | .853 | -.03471 | .09283 |
|  | Experimental group 4 | Control group 4 | .099063^*^ | .020670 | .000 | .03529 | .16283 |

|  |  |  | Mean Difference (I-J) | Std. Error | Sig. | 95% Confidence Interval | |
| --- | --- | --- | --- | --- | --- | --- | --- |
|  |  |  |  |  |  | Lower Bound | Upper Bound |
| Tukey HSD | Experimental group 1 | Control group 1 | -.032625 | .014449 | .326 | -.07720 | .01195 |
|  | Experimental group 2 | Control group 2 | -.008188 | .014449 | .999 | -.05277 | .03639 |
|  | Experimental group 3 | Control group 3 | .042187 | .014449 | .078 | -.00239 | .08677 |
|  | Experimental group 4 | Control group 4 | .010687 | .014449 | .996 | -.03389 | .05527 |

Table S 8: Tukey pairwise post-hoc comparison between the growth rate of the control of Balb/c 3T3 mouse fibroblast cells and Ch-Np showing no statistical difference between the groups.
